# Double-balloon endoscopy-assisted bile duct biopsy with a long, tapered delivery system

**DOI:** 10.1055/a-2779-3500

**Published:** 2026-02-03

**Authors:** Yuki Oka, Arata Sakai, Norimitsu Uza, Yuzo Kodama

**Affiliations:** 1 12885 Division of Gastroenterology, Department of Internal Medicine Kobe, Kobe University, Kobe, Japan

**Fig. 1:**
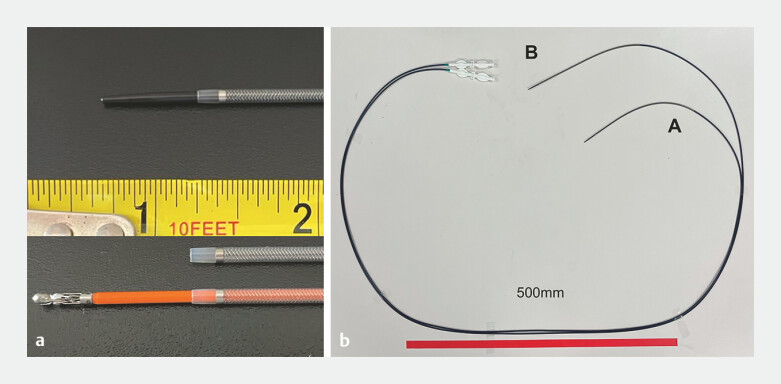
**a** EndoSheather consists of a coaxial dual-layer structure composed of an inner catheter and an outer sheath. Radiopaque markers provide excellent fluoroscopic visibility, and the minimal caliber difference between the inner and outer components ensures high crossing performance through tight biliary strictures. The system allows the smooth insertion of various accessories. **b** Comparison of the novel EndoSheather-long (B) with the conventional EndoSheather (A). Although both have the same inner (2.06 mm) and outer (2.44 mm) sheath diameters, the effective length is extended from 1,707 mm to 1,900 mm, enabling use with long DBE scopes. The scale bar is 500 mm. DBE, double-balloon endoscopy.

**Fig. 2:**
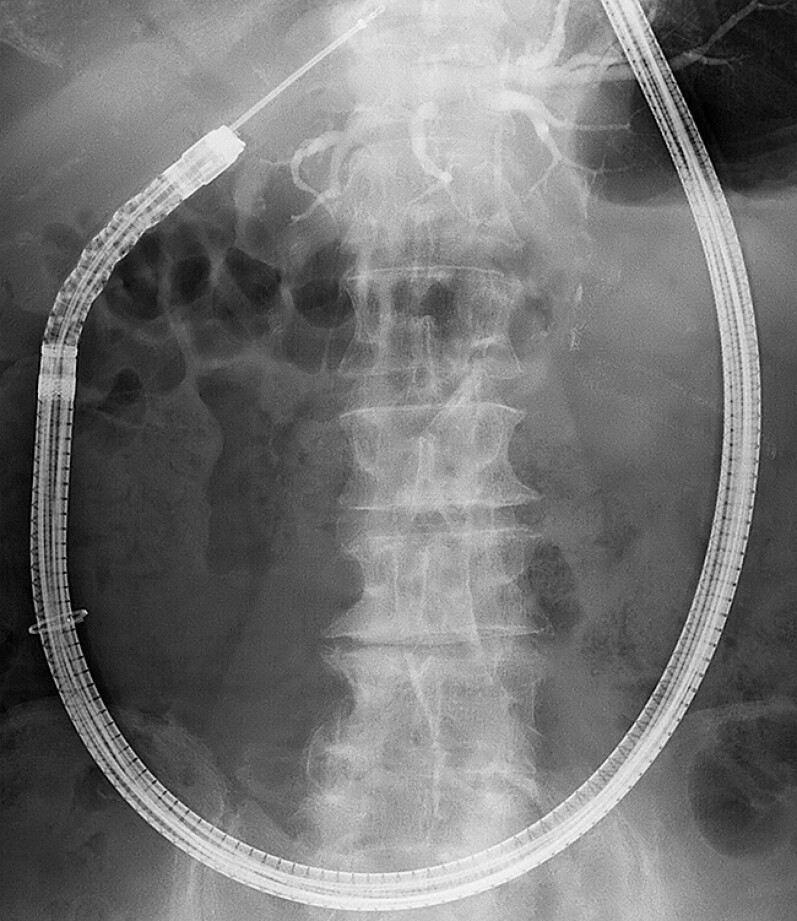
A fluoroscopic image showing the device advanced through DBE to the hepaticojejunostomy. EndoSheather-long traverses the left hepatic duct stricture while maintaining a stable endoscopic position. DBE, double-balloon endoscopy.

Advances in chemotherapy have prolonged survival in cholangiocarcinoma, making postoperative biliary recurrence increasingly common; re-resection for recurrence is undertaken in selected cases. Conventional endoscopic retrograde cholangiopancreatography (ERCP)-based sampling for malignant biliary strictures has the only modest sensitivity (approximately 48% 1 ). Although a tapered delivery system has been reported to aid diagnosis of biliary strictures 2 , no adjunct has been available to support forceps biopsy through long endoscopes such as double-balloon endoscopes (DBEs). Moreover, in surgically altered anatomy, biliary biopsy has been identified as an independent risk factor for ERCP-related adverse events 3 , underscoring the need for safer, more reliable techniques. We describe a novel long, tapered delivery system (EndoSheather-long; Piolax, Kanagawa, Japan) compatible with DBE that preserves a coaxial alignment over a guidewire, reduces friction, and stabilizes the approach across sharp angulation, enabling controlled biopsy in altered anatomy and potentially improving both the diagnostic yield and the safety ( Fig. 1 and Fig. 2 ).

**Fig. 3:**
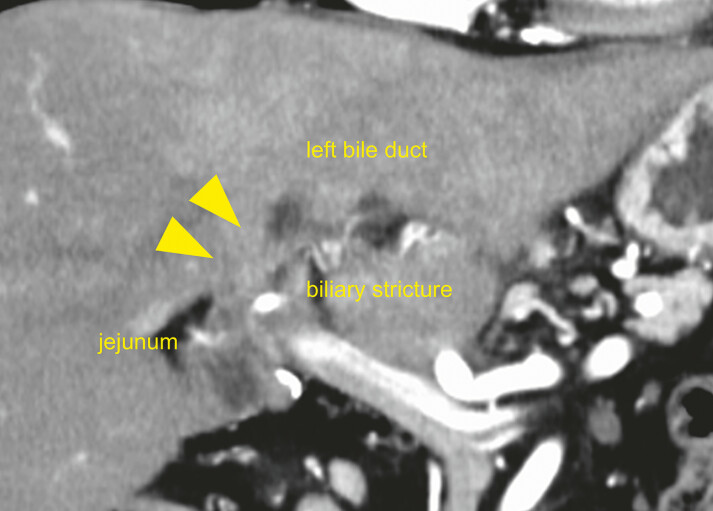
A contrast-enhanced CT image demonstrating a biliary stricture at the hepaticojejunostomy site (arrowheads), with upstream dilatation of the left bile duct. CT, computed tomography.

Two years earlier, a 67-year-old man had undergone subtotal stomach-preserving pancreaticoduodenectomy for distal cholangiocarcinoma. At a routine follow-up, liver enzymes were elevated, and contrast-enhanced computed tomography demonstrated a left hepatic duct stricture with upstream biliary dilatation ( Fig. 3 , Video 1 ).

Double-balloon endoscopy (DBE)-assisted biopsy of a postoperative biliary stricture using a long, tapered delivery system (EndoSheather-long). This system maintains coaxial alignment across acute angulation and enables controlled forceps biopsy in surgically altered anatomy.Video 1

**Fig. 4:**
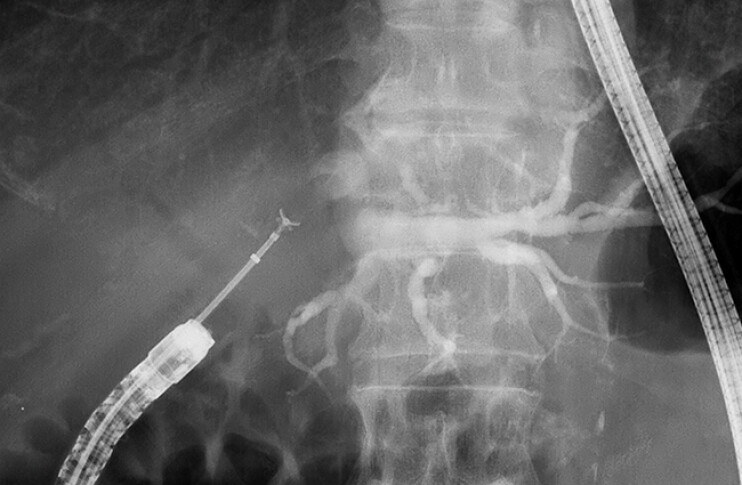
A fluoroscopic image showing bile duct biopsy of the biliary stricture performed through EndoSheather-long using DBE. The stable scope position allowed multiple biopsies to be safely obtained from the stricture site. DBE, double-balloon endoscopy.

**Fig. 5:**
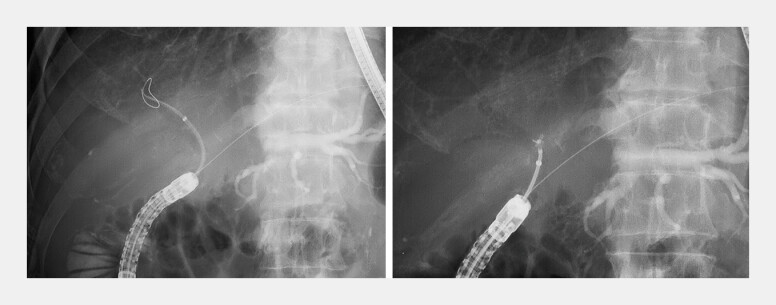
EndoSheather-long could be easily advanced even into the markedly angulated right bile duct, enabling safe and straightforward access for bile duct biopsy.

DBE was advanced to the hepaticojejunostomy. The left hepatic duct was selectively cannulated, and cholangiography showed a 30-mm stricture with upstream dilatation. EndoSheather-long was passed across the lesion, allowing multiple forceps biopsies, while a stable endoscope position was maintained ( Fig. 4 ). A guidewire was then left in the right hepatic duct and the device was reintroduced; despite the acute takeoff angle, it tracked smoothly and enabled a negative biopsy ( Fig. 5 ). Histopathology from the left-duct stricture confirmed adenocarcinoma.

In surgically altered anatomy accessible only by long scopes (e.g., DBE), EndoSheather-long may provide coaxial, stable access for controlled bile duct biopsy, potentially improving the safety and diagnostic yield.

Endoscopy_UCTN_Code_TTT_1AR_2AK
